# Machine Perfusion and Oncologic Outcomes After Liver Transplantation for Hepatocellular Carcinoma: A Systematic Review and Meta-Analysis

**DOI:** 10.3390/curroncol33100578

**Published:** 2026-09-25

**Authors:** Seoung Hoon Kim, Byeong Ho An, Jin A Lee, Go Woon Jeong

**Affiliations:** Organ Transplantation Center, National Cancer Center, Korea, 323 Ilsan-ro, Ilsandong-gu, Goyang-si 10408, Gyeonggi-do, Republic of Korea; 70938@ncc.re.kr (B.H.A.); jina71@ncc.re.kr (J.A.L.); 11821@ncc.re.kr (G.W.J.)

**Keywords:** hepatocellular carcinoma, liver transplantation, machine perfusion, hypothermic oxygenated perfusion, recurrence-free survival, tumor recurrence, ischemia–reperfusion injury, transplant oncology

## Abstract

Background: Machine perfusion has emerged as a promising strategy to attenuate ischemia–reperfusion injury during liver transplantation (LT). However, its impact on hepatocellular carcinoma (HCC) recurrence and post-transplant oncologic outcomes remains uncertain. We performed a systematic review and meta-analysis evaluating the association between machine perfusion and oncologic outcomes after LT for HCC. Methods: A systematic literature search of PubMed/MEDLINE, Embase, and Web of Science was conducted from database inception to May 2026. Comparative clinical studies evaluating machine perfusion and oncologic outcomes after LT for HCC were included. The primary outcome was recurrence-free survival (RFS). Secondary outcomes included HCC recurrence, early recurrence, overall survival (OS), and graft-related outcomes. Risk of bias was assessed using the ROBINS-I tool. Random-effects meta-analyses were performed when appropriate. Results: Six comparative clinical studies involving HCC recipients undergoing LT were included. All studies were observational, and no randomized controlled trial was identified. Three studies were judged to have a moderate risk of bias and three were judged to have a serious risk of bias. In the primary RFS meta-analysis, machine perfusion was associated with a numerically lower risk of recurrence or death, although the pooled estimate did not reach statistical significance (HR 0.34, 95% CI 0.07–1.77; *p* = 0.20). The binary recurrence analysis also demonstrated a numerically lower risk of HCC recurrence after machine perfusion (RR 0.40, 95% CI 0.13–1.20; *p* = 0.10). Several studies reported favorable associations between machine perfusion and early recurrence, while large adjusted cohorts reported associations with improved RFS and OS. Machine perfusion was generally associated with lower markers of ischemia–reperfusion injury, including postoperative aminotransferase release. Conclusions: Current evidence suggests a potential favorable association between machine perfusion and oncologic outcomes after LT for HCC. However, the pooled estimates from the primary analyses were not statistically significant, and the evidence remains limited by the small number of studies, observational designs, heterogeneity, and potential cohort overlap. Further prospective studies are required before an oncologic benefit of machine perfusion can be established.

## Simple Summary

Liver transplantation is an important curative treatment for selected patients with hepatocellular carcinoma, but cancer recurrence after transplantation remains a major clinical problem. Machine perfusion is a newer method of preserving donor livers before transplantation and may reduce ischemia–reperfusion injury, inflammation, and early graft damage. Because these biological processes may contribute to tumor recurrence, machine perfusion could potentially influence oncologic outcomes after transplantation for hepatocellular carcinoma. In this systematic review and meta-analysis, we summarized the available clinical evidence on machine perfusion and post-transplant cancer outcomes. Six comparative studies were included. Overall, machine perfusion showed a favorable trend toward lower recurrence and better recurrence-free survival, but the pooled results were not statistically significant. The evidence remains limited because all included studies were observational and differed in donor type, perfusion method, follow-up duration, and adjustment for tumor biology. Further prospective randomized studies are needed to determine whether machine perfusion can reduce hepatocellular carcinoma recurrence after liver transplantation.

## 1. Introduction

Liver transplantation (LT) is a potentially curative treatment for selected patients with hepatocellular carcinoma (HCC), as it removes both the tumor and the underlying cirrhotic liver that provides a carcinogenic microenvironment. Since the introduction of the Milan criteria, refinements in candidate selection incorporating tumor morphology, alpha-fetoprotein, radiologic response to locoregional therapy, and other biological surrogates have improved post-transplant outcomes [1,2]. Nevertheless, HCC recurrence after LT remains a major clinical problem and is one of the strongest determinants of long-term survival. Once recurrence develops, treatment options are often limited by immunosuppression, graft function, extrahepatic disease burden, and aggressive tumor biology [2,3].

The risk of post-transplant HCC recurrence has traditionally been attributed mainly to tumor-related factors, including tumor size and number, alpha-fetoprotein (AFP) level, tumor differentiation, microvascular invasion, response to bridging or downstaging therapy, and fulfillment of established transplant criteria [1,2,3]. However, recurrence may also be influenced by perioperative and graft-related factors. In particular, ischemia time and ischemia–reperfusion injury (IRI) have gained attention as potentially modifiable contributors to HCC recurrence after LT [4,5]. IRI is characterized by mitochondrial dysfunction, oxidative stress, endothelial activation, Kupffer cell activation, neutrophil recruitment, cytokine release, and hepatocellular injury. These processes may create a pro-inflammatory and pro-angiogenic microenvironment that facilitates the survival and outgrowth of residual circulating tumor cells or occult micrometastases [5,6,7,8].

Machine perfusion (MP) has emerged as an important strategy for liver graft preservation and reconditioning. In contrast to static cold storage (SCS), MP allows controlled perfusate circulation, oxygen delivery, metabolic support, and, in some platforms, viability assessment before implantation. Hypothermic oxygenated perfusion (HOPE), dual hypothermic oxygenated perfusion (D-HOPE), hypothermic machine perfusion (HMP), and normothermic machine perfusion (NMP) have been associated with reduced biochemical graft injury, improved early graft function, and fewer ischemia-related complications in selected LT settings [9,10,11,12,13]. Although these benefits have primarily been evaluated in relation to graft outcomes, MP may also have oncologic implications in recipients transplanted for HCC by attenuating IRI-related inflammatory signaling and recurrence-promoting microenvironmental changes [14,15,16].

Clinical evidence regarding the association between MP and oncologic outcomes after LT for HCC remains limited and heterogeneous. An early study suggested that HOPE might be associated with reduced HCC recurrence and improved recurrence-free survival (RFS), particularly in recipients of donation-after-circulatory-death (DCD) grafts [17]. In contrast, another study reported reductions in biochemical markers of graft injury without a clear improvement in HCC recurrence or RFS [18]. More recent propensity-weighted, registry-based, and international matched cohort studies have further examined MP in relation to HCC recurrence, early recurrence, RFS, and overall survival (OS) [19,20,21,22]. However, these studies differ substantially in perfusion modality, donor type, recipient selection, tumor biology, comparator groups, adjustment methods, and follow-up duration.

A previous systematic review evaluated interventions intended to decrease allograft IRI in patients undergoing LT for HCC and identified only four retrospective studies involving 938 patients [23]. Importantly, that review included heterogeneous interventions, such as pharmacologic IRI attenuation, MP, and ischemia-free transplantation, and quantitative synthesis was not performed because of substantial clinical and methodological heterogeneity. Since then, several additional MP-specific clinical studies have been published, providing a timely rationale for an evidence synthesis focused specifically on MP and oncologic outcomes after LT for HCC [19,20,21,22].

Therefore, this study aimed to perform a systematic review and meta-analysis evaluating the association between MP and oncologic outcomes after LT for HCC, with RFS as the primary outcome. Secondary oncologic and graft-related outcomes were also assessed to explore the relationship between MP, IRI, and post-transplant cancer outcomes. By integrating clinical evidence with the biological rationale linking graft preservation, IRI modulation, inflammatory signaling, and tumor recurrence, this review seeks to clarify the current evidence base and identify key gaps for future prospective studies.

## 2. Methods

### 2.1. Protocol and Reporting

This systematic review and meta-analysis was conducted in accordance with the Preferred Reporting Items for Systematic Reviews and Meta-Analyses statement (PRISMA 2020) [24]. The completed PRISMA 2020 checklist is provided in the Supplementary Materials. The study protocol was registered in the International Prospective Register of Systematic Reviews (PROSPERO; registration number: CRD420261406720). Because this study used data from previously published studies, institutional review board approval and informed consent were not required.

### 2.2. Eligibility Criteria

Studies were selected according to the population, intervention, comparator, outcome, and study design framework.

The population of interest was adult patients undergoing LT for HCC. Studies including mixed indications for LT were eligible only if HCC-specific oncologic outcomes were reported separately or could be extracted from the available data.

The intervention of interest was ex situ MP-based liver graft preservation or reconditioning before transplantation, including HOPE, D-HOPE, HMP, and NMP. Studies evaluating combined strategies involving ex situ MP after normothermic regional perfusion were considered eligible if oncologic outcomes after LT for HCC were reported and the MP component was clearly described.

The comparator was conventional static cold storage, unperfused graft preservation, or standard LT without machine perfusion-based graft preservation or reconditioning.

The primary outcome was RFS after LT for HCC. Secondary oncologic outcomes included overall HCC recurrence, early HCC recurrence, OS, disease-free survival, and tumor-free survival. Secondary graft-related and IRI-related outcomes included graft survival, early allograft dysfunction, postoperative peak aminotransferase levels, biliary complications, and other reported markers of IRI.

Randomized controlled trials, prospective cohort studies, retrospective cohort studies, propensity score-matched studies, inverse probability of treatment-weighted studies, registry-based studies, and other comparative clinical studies were eligible. Non-comparative studies, case reports, editorials, letters without original data, narrative reviews, systematic reviews, conference abstracts without sufficient extractable data, animal studies, and in vitro studies were excluded from the primary analysis. Studies evaluating non-MP interventions for IRI, such as pharmacologic interventions, were excluded from the primary analysis. Ischemia-free LT studies were considered mechanistically relevant but were not included in the primary quantitative synthesis because this strategy differs conceptually from conventional ex situ MP versus SCS comparisons.

### 2.3. Information Sources and Search Strategy

A systematic literature search was performed in PubMed/MEDLINE, Embase, and Web of Science Core Collection from database inception to 31 May 2026. The search was restricted to articles published in English, with no restriction on publication year.

The reference lists of included studies and relevant reviews were manually screened. In addition, supplementary manual searches of Google Scholar, relevant journal websites, and publisher platforms were performed to identify recently published full-text articles that might not yet have been indexed in PubMed/MEDLINE at the time of the final search.

The search strategy combined terms related to LT, HCC, and MP. Terms related to RFS, tumor recurrence, disease-free survival, and OS were not included as mandatory search concepts in the main database strategies to maximize sensitivity. These outcomes were assessed during title/abstract screening, full-text review, and data extraction.

The complete search strategies for all databases are provided in Supplementary Table S1.

### 2.4. Study Selection

All retrieved records were imported into EndNote, and duplicates were removed. Two reviewers independently screened titles and abstracts according to the predefined eligibility criteria. Potentially eligible studies were then assessed by full-text review. Disagreements were resolved through discussion, and unresolved discrepancies were adjudicated by a third reviewer.

When multiple reports appeared to include overlapping patient cohorts, the most comprehensive, most recent, or methodologically robust study was prioritized for the primary analysis. Potential cohort overlap was assessed by comparing study institutions, data sources, study periods, sample sizes, intervention groups, and author lists. Potential cohort overlap was considered likely when studies shared participating institutions or registries, substantially overlapping recruitment periods, similar machine-perfusion populations or comparator groups, and overlapping investigator groups. When overlap could not be confidently excluded, the most comprehensive, recent, or methodologically robust dataset was retained in the primary analysis, whereas potentially overlapping datasets were evaluated only in sensitivity analyses.

### 2.5. Data Extraction

Two reviewers independently extracted data using a standardized data extraction form. Extracted information included first author, publication year, country, study design, study period, data source, number of centers, sample size, MP modality, comparator, donor type, graft type, recipient characteristics, tumor characteristics, Milan criteria status, AFP level, locoregional therapy, bridging or downstaging therapy, cold ischemia time, warm ischemia time, perfusion duration, follow-up duration, immunosuppression when reported, and adjustment methods.

For outcomes, we extracted hazard ratios (HRs) with 95% confidence intervals (CIs) for RFS, disease-free survival, tumor-free survival, and OS whenever available. For binary outcomes, we extracted event counts and total numbers for HCC recurrence, early recurrence, graft loss, early allograft dysfunction, biliary complications, and postoperative complications. For continuous outcomes, including peak postoperative aspartate aminotransferase (AST) and alanine aminotransferase (ALT) levels, data were extracted as reported in the original studies. Although conversion of medians and interquartile ranges to means and standard deviations was considered when necessary, no converted continuous outcome data were used in the quantitative meta-analyses because graft-related continuous outcomes were too heterogeneous for meaningful pooling. These outcomes were therefore summarized qualitatively. For time-to-event outcomes, adjusted HRs with 95% CIs were preferentially extracted. If adjusted estimates were unavailable, unadjusted HRs were extracted. When HRs were not directly reported, they were estimated from Kaplan–Meier curves using previously described methods if sufficient data were available [25].

Adjusted effect estimates were preferentially extracted over unadjusted estimates. For propensity score-matched, inverse probability of treatment-weighted, or multivariable-adjusted analyses, the variables included in the adjustment model were recorded.

### 2.6. Outcomes

The primary outcome was RFS after LT for HCC. RFS was defined according to each study’s definition and generally represented the interval from LT to HCC recurrence or death.

Secondary oncologic outcomes included overall HCC recurrence, early HCC recurrence, disease-free survival, tumor-free survival, OS, and cancer-specific survival when reported. Early recurrence was defined according to the definition used in each included study. When sufficiently comparable data were available, recurrence occurring within 2 years after transplantation was used as an operational definition of early recurrence, because most post-transplant HCC recurrences occur during the first 2 years and recurrence within this interval has been associated with more aggressive tumor biology and poorer prognosis [26]. However, study-specific definitions were retained when explicitly reported, and no uniform threshold was retrospectively imposed across studies.

Secondary graft-related and IRI-related outcomes included graft survival, early allograft dysfunction, peak postoperative AST and ALT levels, biliary complications, ischemic cholangiopathy, and other inflammatory or biochemical markers of graft injury when available.

### 2.7. Risk-of-Bias Assessment

The risk of bias of non-randomized comparative studies was assessed independently by two reviewers using the Risk Of Bias In Non-randomized Studies of Interventions tool (ROBINS-I) [27]. The following domains were evaluated: bias due to confounding, selection of participants, classification of interventions, deviations from intended interventions, missing data, measurement of outcomes, and selection of the reported result. Each study was judged as having low, moderate, serious, critical risk of bias, or no information.

### 2.8. Data Synthesis and Statistical Analysis

A qualitative synthesis was first performed to summarize study characteristics, patient populations, perfusion modalities, donor types, oncologic outcomes, graft-related outcomes, and risk of bias. Quantitative meta-analysis was performed when at least two studies reported sufficiently comparable outcomes.

For time-to-event outcomes, HRs were pooled using the generic inverse variance method. HRs and 95% CIs were log-transformed before analysis.

For binary outcomes, risk ratios (RRs) with 95% CIs were calculated. Odds ratios (ORs) were considered in sensitivity analyses when event rates were low. For studies with zero events in one treatment group, a continuity correction was applied.

Random-effects meta-analyses were performed using the DerSimonian–Laird method-of-moments estimator for the between-study variance (τ^2^) [28]. Study-specific log-transformed effect estimates were weighted using inverse-variance weights incorporating both within-study variance and τ^2^. The pooled effect estimate and 95% CI were calculated on the logarithmic scale and subsequently exponentiated to obtain pooled HRs or RRs. Statistical heterogeneity was assessed using Cochran’s Q test and the I^2^ statistic, with I^2^ values of approximately 25%, 50%, and 75% interpreted as low, moderate, and high heterogeneity, respectively. For binary outcomes with zero events in one study arm, a continuity correction of 0.5 was applied to both groups of that study before calculation of the RR.

Publication bias and small-study effects were not formally assessed because fewer than 10 studies were available for each pooled outcome; funnel plots and Egger’s test were prespecified only when at least 10 studies were available. All statistical analyses were performed using R software (version 4.3.2; R Foundation for Statistical Computing, Vienna, Austria) with the meta and metafor packages. A two-sided *p* value of < 0.05 was considered statistically significant. The study-level data used for the meta-analyses and sensitivity analyses are provided in Supplementary Dataset S1.

### 2.9. Subgroup and Sensitivity Analyses

Prespecified subgroup analyses were planned according to machine perfusion modality, including HOPE, D-HOPE, HMP, and NMP; donor type, including donation after brain death (DBD) and DCD; study design; adjustment method; and recurrence timing. If sufficient data were available, additional subgroup analyses were planned according to Milan criteria status, AFP level, and tumor burden.

Sensitivity analyses were planned by excluding studies at serious or critical risk of bias, excluding studies with potentially overlapping cohorts, excluding registry-based studies, excluding studies involving combined normothermic regional perfusion and ex situ MP strategies, and restricting the analysis to propensity score-matched or adjusted studies.

Because the anticipated number of included studies was small, subgroup analyses were considered exploratory and interpreted cautiously.

## 3. Results

### 3.1. Study Selection

A total of 195 records were identified through the systematic literature search. Of these, 181 records were retrieved from the three electronic databases, including PubMed/MEDLINE (n = 38), Embase (n = 82), and Web of Science Core Collection (n = 61). An additional 14 records were identified through supplementary manual searches of reference lists, Google Scholar, journal websites, and publisher platforms. After removing 72 duplicate records, 123 records were screened by title and abstract. Among them, 94 records were excluded because they were not relevant to the review question.

The full texts of 29 reports were retrieved and assessed for eligibility. No reports were unavailable for full-text review. After full-text assessment, 23 reports were excluded for the following reasons: absence of HCC-specific oncologic outcomes (n = 6), absence of a comparative machine perfusion group (n = 4), evaluation of non-MP interventions for IRI (n = 1), ischemia-free LT or other non-standard ischemia-reduction strategies outside the primary analysis scope (n = 2), insufficient extractable data or conference abstract only (n = 5), review, editorial, or protocol without eligible clinical outcome data (n = 4), and overlapping cohort data prioritized for exclusion (n = 1).

Finally, six full-text comparative clinical studies were included in the qualitative synthesis, and all six studies were included in the quantitative synthesis. Because data availability differed by oncologic and graft-related outcomes, and potentially overlapping cohorts were handled on an outcome-specific basis, not all six studies contributed to every meta-analysis. The study selection process is summarized in Figure 1.

### 3.2. Study Characteristics

Six full-text comparative clinical studies were included in the qualitative and quantitative syntheses. The studies were published between 2020 and 2026 and included adult recipients undergoing LT for HCC. All included studies were non-randomized comparative studies, consisting of retrospective cohort studies, propensity score-adjusted or matched analyses, registry-based analyses, and multicenter observational matched cohort analyses. No randomized controlled trial directly evaluating machine perfusion and oncologic outcomes after LT for HCC was identified.

The included studies evaluated different MP strategies. HOPE was the most frequently studied modality and was assessed either as end-ischemic HOPE or as part of HOPE-based preservation strategies. D-HOPE was evaluated in one single-center study and was also included within the HOPE-REAL cohort. One registry-based study evaluated MP more broadly using the UNOS database, in which the specific MP modality was not fully specified. Across studies, the comparator was SCS, non-perfused graft preservation, or conventional LT without ex situ MP.

The study populations differed substantially in donor type, perfusion indication, comparator structure, and analytic approach. Mueller et al. evaluated HCC recurrence after transplantation using HOPE-treated DCD grafts and compared outcomes with non-perfused control grafts [17]. Rigo et al. compared 80 recipients of D-HOPE-treated grafts with 246 recipients of SCS-preserved grafts in a single-center cohort of 326 HCC recipients [18]. Dajti et al. analyzed 237 consecutive HCC recipients and evaluated the association of cold ischemia time and HOPE with early HCC recurrence using propensity score weighting [19]. Shu et al. used the UNOS database and, after 1:1 propensity score matching, compared 411 recipients in the MP group with 411 recipients in the SCS group [20]. Eden et al. performed a post hoc analysis of the multicenter HOPE-REAL study and included 599 HCC recipients receiving HOPE-treated grafts, with matched comparisons against non-HCC recipients and an external non-perfused HCC control cohort [21]. Koch et al. evaluated 137 HCC recipients transplanted with DBD grafts and compared 32 HOPE recipients with 105 non-HOPE controls using propensity score matching [22].

The primary oncologic endpoint varied across studies but generally included RFS, HCC recurrence, early recurrence, tumor-free survival, or overall survival. In addition to oncologic outcomes, several studies reported graft-related outcomes, including early allograft dysfunction, postoperative complications, biliary complications, and biochemical markers of IRI such as postoperative AST and ALT peaks. The degree of adjustment for confounding differed across studies, ranging from multivariable regression and inverse probability of treatment weighting to propensity score matching. Key characteristics of the included studies are summarized in Table 1, and their main oncologic outcomes are summarized in Table 2.

Additional center-, donor-, tumor-, and perioperative characteristics were inconsistently reported across the included studies. Information regarding donor source, use of normothermic regional perfusion, HCC selection criteria, pretransplant treatment, waiting time, postoperative complications, intensive care unit and hospital stay, and immunosuppression is summarized in Supplementary Table S2.

### 3.3. Risk of Bias Assessment

The risk of bias assessment is summarized in Figure 2 and Table 3. No randomized controlled trials were identified; therefore, all included studies were evaluated using the ROBINS-I tool for non-randomized studies of interventions.

Overall, three studies were judged to have a moderate risk of bias and three were judged to have a serious risk of bias. The main sources of bias were confounding and participant selection, reflecting the non-randomized allocation of MP, its preferential use in DCD or extended-criteria donor grafts, center-specific practice patterns, temporal changes following implementation of MP, and differences in follow-up duration.

Classification of the intervention was generally reliable, although registry-based and multicenter studies occasionally grouped different MP modalities together. Bias in outcome measurement was judged as low to moderate because HCC recurrence and survival are objective outcomes; however, recurrence surveillance protocols and ascertainment may have varied across centers. No study was judged to have a critical risk of bias.

### 3.4. Recurrence-Free Survival

RFS was reported in all six included studies, although the reporting format and availability of effect estimates differed across studies. Directly extractable HRs for RFS were available from four studies. However, because the HOPE-REAL cohort by Eden et al. [21] potentially overlapped with several European single-center HOPE cohorts, it was excluded from the primary pooled analysis and considered in sensitivity or narrative interpretation. Therefore, the primary RFS meta-analysis included three non-overlapping studies with extractable hazard ratios.

In the primary random-effects meta-analysis, MP was associated with a numerically lower risk of recurrence or death, although the pooled estimate did not reach statistical significance (HR 0.34, 95% CI 0.07–1.77; *p* = 0.20; Figure 3). Substantial heterogeneity was observed across studies (I^2^ = 78.4%, τ^2^ = 1.610; *p* for heterogeneity = 0.010). The direction of effect differed across studies: Dajti et al. [19] and Shu et al. [20] reported significantly improved RFS after MP, whereas Rigo et al. [18] found no RFS benefit with D-HOPE.

Among studies not included in the primary pooled RFS analysis, Mueller et al. reported higher 5-year RFS in recipients of HOPE-treated DCD grafts compared with recipients of non-perfused DBD grafts at the same center (92% vs. 73%; *p* = 0.027) [17]. Eden et al., the largest multicenter matched cohort study, also reported improved RFS in HOPE-treated HCC recipients compared with matched recipients of non-perfused grafts, with a multivariable HR of 0.48 (95% CI 0.28–0.83; *p* = 0.008) [21]. In contrast, Koch et al. reported no statistically significant difference in RFS after propensity score matching despite observing no HCC recurrence in the HOPE group during the comparable follow-up period [22].

Overall, the available evidence suggests a possible favorable association between MP and RFS after LT for HCC. However, the certainty of this finding is limited by the small number of studies, observational study designs, substantial heterogeneity, differences in perfusion modality and donor type, and potential cohort overlap among European HOPE cohorts. Sensitivity analysis incorporating the potentially overlapping HOPE-REAL cohort yielded a pooled HR of 0.41 (95% CI 0.16–1.03; I^2^ = 67.7%), compared with 0.34 (95% CI 0.07–1.77; I^2^ = 78.4%) in the primary analysis, indicating that inclusion of HOPE-REAL did not materially alter the direction of the association (Supplementary Figure S1).

### 3.5. HCC Recurrence

HCC recurrence was reported in all six included studies, although group-specific event counts were not consistently available for quantitative pooling. Four non-overlapping studies provided extractable recurrence event counts and were included in the primary binary recurrence meta-analysis. For studies reporting early recurrence or recurrence within a comparable follow-up period, the corresponding event counts were used for this analysis.

In the primary event-count analysis, HCC recurrence occurred in 13 of 242 recipients in the MP group and in 62 of 598 recipients in the control group. Individual study results were directionally favorable for MP in three studies, whereas one study reported comparable recurrence rates between D-HOPE and SCS. Mueller et al. reported recurrence in 4/70 recipients of HOPE-treated DCD grafts compared with 18/70 recipients of non-perfused DBD grafts [17]. Rigo et al. reported comparable recurrence rates between D-HOPE and SCS groups (8/80 vs. 22/246) [18]. Dajti et al. reported lower 2-year recurrence in the HOPE group compared with SCS (1/60 vs. 14/177) [19]. Koch et al. reported no recurrence in the HOPE group and 8/105 recurrences in the control group within a comparable follow-up period [22].

Random-effects meta-analysis showed a numerically lower risk of HCC recurrence after MP, but the pooled estimate did not reach statistical significance (RR 0.40, 95% CI 0.13–1.20; *p* = 0.10; Figure 4). Moderate-to-substantial heterogeneity was observed across studies (I^2^ = 60.8%, τ^2^ = 0.700; *p* for heterogeneity = 0.054). The result was driven by more favorable estimates in Mueller et al. and Dajti et al., [17,19] whereas the neutral result of Rigo et al. attenuated the pooled estimate [18].

Eden et al. was not included in the primary event-count meta-analysis because the HOPE-REAL cohort may overlap with several European single-center HOPE cohorts. However, in its external matched comparison, HCC recurrence was lower among recipients of HOPE-treated grafts than among recipients of non-perfused grafts (23/312 [7.4%] vs. 59/312 [18.9%]; *p* < 0.0001), supporting the direction of the primary analysis [21]. Shu et al. was also not included in the crude event-count meta-analysis because raw recurrence counts were not directly extractable from the main text; however, the study reported lower 2-year recurrence in the MP group using time-to-event and competing-risk analyses [20].

Overall, the binary recurrence analysis suggested a potential reduction in HCC recurrence after MP, but this finding should be interpreted cautiously because of the small number of studies, heterogeneous recurrence definitions, differences in follow-up duration, zero-event correction in one study, and possible cohort overlap among HOPE-treated European cohorts. Sensitivity analysis incorporating the HOPE-REAL cohort yielded a pooled RR of 0.42 (95% CI 0.22–0.83; I^2^ = 52.8%), compared with 0.40 (95% CI 0.13–1.20; I^2^ = 60.8%) in the primary analysis. Although the sensitivity estimate reached statistical significance, it was interpreted cautiously because potential cohort overlap could not be excluded (Supplementary Figure S2).

Publication bias and small-study effects were not formally assessed because fewer than 10 studies were available for each pooled analysis.

### 3.6. Early HCC Recurrence

Early HCC recurrence was specifically evaluated in three studies, although definitions and statistical approaches differed. Dajti et al. defined early recurrence as recurrence occurring within the first 2 years after transplantation. In that cohort, early recurrence occurred in 15 of 237 recipients overall, with a median time to recurrence of 13.4 months. The 2-year recurrence rate was lower in the HOPE group than in the SCS group (1/60 [2%] vs. 14/177 [8%]; *p* = 0.10). After propensity score weighting, HOPE was associated with a significantly lower risk of early recurrence (weighted OR 0.126, 95% CI 0.016–0.989; *p* = 0.049) [19].

Shu et al. also focused on early post-transplant tumor recurrence in a large UNOS-based cohort. After propensity score matching, the 2-year RFS rate was significantly higher in the MP group than in the SCS group (96.5% vs. 89.7%; *p* = 0.008). In competing-risk analysis, the 2-year cumulative recurrence rate was lower in the MP group than in the SCS group (1.7% vs. 9.3%; *p* = 0.006), and MP remained protective in the Fine–Gray subdistribution hazard model (adjusted HR 0.172, 95% CI 0.047–0.625; *p* = 0.008) [20].

Koch et al. evaluated recurrence within a comparable early follow-up period because the HOPE group had a shorter follow-up duration than the no-HOPE group. No recurrence was observed among 32 HOPE recipients during a median follow-up of 771 days, whereas 8 of 105 control recipients (7.6%) developed recurrence within the same time frame. After propensity score matching, time-to-recurrence differed significantly between the HOPE and no-HOPE groups (*p* = 0.032), although RFS and OS did not differ significantly [22].

Because early recurrence was reported using different definitions and effect measures, a separate pooled analysis for early recurrence was not performed. Nevertheless, all three studies evaluating early recurrence showed directionally favorable results for MP, supporting the hypothesis that attenuation of IRI may be most relevant during the early post-transplant period. These findings suggest a potential association between MP and reduced early HCC recurrence, but the evidence remains limited by retrospective study designs, heterogeneous definitions, and residual confounding.

### 3.7. Overall Survival

OS was reported inconsistently across the included studies and was not suitable for a dedicated pooled analysis. Direct adjusted effect estimates for OS were available from two studies, whereas other studies either did not report OS as a comparative time-to-event outcome or reported only non-significant Kaplan–Meier comparisons.

In the UNOS-based propensity score-matched cohort by Shu et al., OS was significantly higher among recipients of machine-perfused grafts than among recipients of SCS grafts. In multivariable Cox regression, MP was independently associated with improved OS, with an adjusted HR of 0.316 (95% CI 0.160–0.627; *p* < 0.001). In subgroup analyses, the OS benefit appeared more evident in the predefined beneficial subgroup, whereas no significant OS difference was observed in the non-beneficial subgroup [20].

Eden et al. reported favorable long-term survival in the HOPE-REAL HCC cohort. In the overall HOPE-treated HCC cohort, 1-, 3-, and 5-year OS rates were 92%, 86%, and 81%, respectively. After propensity score matching, 5-year OS was comparable between HOPE-treated HCC recipients and matched non-HCC recipients (82% vs. 84%; *p* = 0.625). In contrast, in the external matched DBD comparison, 5-year OS was significantly higher among HOPE-treated HCC recipients than among recipients of non-perfused grafts (84% vs. 74%; *p* = 0.034). Multivariable Cox regression also identified HOPE as independently associated with improved OS (HR 0.64, 95% CI 0.43–0.95; *p* = 0.030) [21].

In contrast, Koch et al. did not observe a statistically significant OS difference between HOPE and no-HOPE groups. In the unmatched cohort, OS did not differ significantly between groups (log-rank *p* = 0.820), and this remained non-significant after propensity score matching (*p* = 0.246) [22]. Similarly, Rigo et al. did not demonstrate an OS advantage with D-HOPE; the estimated 5-year survival probability based on the Metroticket 2.0 model was comparable between D-HOPE and SCS groups [18]. Mueller et al. and Dajti et al. primarily focused on recurrence-related outcomes, and comparative OS estimates were not available for quantitative synthesis [17,19].

Overall, the available OS data suggest a possible survival advantage associated with MP in large adjusted cohorts, but the evidence remains insufficient for definitive conclusions because OS was inconsistently reported and was strongly influenced by graft-, recipient-, and center-level factors beyond HCC recurrence.

### 3.8. Graft-Related Outcomes and Markers of Ischemia–Reperfusion Injury

Graft-related outcomes and biochemical markers of IRI were reported inconsistently across the included studies; therefore, these endpoints were summarized qualitatively. The most commonly reported markers were postoperative aminotransferase peaks, early allograft dysfunction, biliary complications, and acute rejection.

Several studies suggested that MP was associated with attenuation of biochemical graft injury. In Rigo et al., peak AST and ALT levels were significantly lower in the D-HOPE group than in the SCS group (AST: 903 vs. 1140 U/L, *p* = 0.022; ALT: 496.5 vs. 742 U/L, *p* = 0.002), although this biochemical improvement did not translate into lower HCC recurrence or improved RFS. Early allograft dysfunction was comparable between groups (26% vs. 27%; *p* = 1.000), as were overall biliary complications (18% vs. 20%; *p* = 0.754) and ischemic cholangiopathy (0% vs. 3%; *p* = 0.280) [18].

Dajti et al. also reported numerically lower aminotransferase peaks and early allograft dysfunction in the HOPE group, although these differences were not statistically significant. Peak ALT was 492 U/L in the HOPE group and 623 U/L in the SCS group (*p* = 0.1), peak AST was 508 U/L and 646 U/L, respectively (*p* = 0.2), and early allograft dysfunction occurred in 13% and 21% of recipients, respectively (*p* = 0.2). Primary non-function and grade ≥3 complications at 90 days were also comparable between groups [19].

Koch et al. reported a marked reduction in peak AST after HOPE treatment in DBD graft recipients. The mean peak AST level within the first 7 days after transplantation was 1444.6 U/L in the HOPE group compared with 3557.4 U/L in the no-HOPE group (*p* < 0.001). However, RFS and OS did not differ significantly between groups, suggesting that lower hepatocellular injury alone may not be sufficient to demonstrate a short-term survival advantage in a small cohort [22].

Mueller et al. reported mixed biochemical findings. Recipients of HOPE-treated DCD grafts had higher day-1 ALT levels than recipients of non-perfused DBD grafts, reflecting the higher-risk DCD setting, but post-transplant C-reactive protein levels were lower in the HOPE-treated group, suggesting reduced systemic inflammation. The same study also reported a lower biopsy-proven acute rejection rate in the HOPE-treated group [17].

In the HOPE-REAL cohort reported by Eden et al., biochemical graft injury markers were presented descriptively within the HOPE-treated HCC cohort rather than as a direct comparison with non-perfused controls. Peak AST and ALT levels were higher in DCD than in DBD grafts, but HCC recurrence rates were similar between DCD and DBD recipients. Therefore, these data support the feasibility of HOPE-treated grafts across different donor types but were not suitable for pooled comparison of graft injury markers against SCS [21].

Overall, MP was generally associated with lower or numerically improved markers of hepatocellular injury in studies that reported these endpoints. However, the relationship between biochemical attenuation of IRI and oncologic benefit was inconsistent. In particular, Rigo et al. demonstrated lower AST and ALT peaks without improved RFS, ref. [18] whereas Dajti et al., Shu et al., and Eden et al. reported favorable oncologic outcomes in adjusted analyses [19,20,21]. These findings suggest that MP may reduce graft injury, but the extent to which this translates into reduced HCC recurrence likely depends on donor type, tumor biology, recurrence risk, perfusion modality, and follow-up duration.

## 4. Discussion

In this systematic review and meta-analysis, we evaluated the association between MP and oncologic outcomes after LT for HCC. Six full-text comparative clinical studies were included. Overall, the available evidence suggests a possible favorable association between MP and post-transplant oncologic outcomes, particularly HCC recurrence and RFS. However, the pooled estimates did not reach statistical significance in the primary analyses, and the certainty of evidence remains limited by observational study designs, heterogeneity in perfusion modality and donor type, differences in follow-up duration, and potential cohort overlap among European HOPE cohorts.

The primary meta-analysis of RFS showed a numerically lower risk of recurrence or death after MP, but the association was not statistically significant. This result should be interpreted in the context of substantial heterogeneity. Dajti et al. and Shu et al. reported favorable associations between MP and RFS, refs. [19,20] whereas Rigo et al. reported no RFS benefit after D-HOPE despite lower postoperative aminotransferase peaks [18]. Dajti et al. reported that, after propensity score weighting, HOPE was associated with both lower early recurrence and higher RFS [19]. In contrast, Rigo et al. found comparable recurrence rates between D-HOPE and SCS and reported an adjusted HR close to null for RFS, despite significantly lower AST and ALT peaks in the D-HOPE group [18]. These divergent findings suggest that biochemical attenuation of IRI may not uniformly translate into measurable oncologic benefit across all clinical settings.

The analysis of binary HCC recurrence also suggested a directionally favorable effect of MP, although the pooled estimate did not reach statistical significance. Recurrence rates were lower in several HOPE-based studies, including Mueller et al., Dajti et al., and Koch et al., [17,19,22] whereas Rigo et al. reported comparable recurrence between D-HOPE and SCS [18]. Koch et al. observed no recurrence among 32 HOPE recipients during a median follow-up of 771 days, compared with recurrence in 8 of 105 no-HOPE recipients within the same comparable follow-up window; however, RFS and OS remained non-significant after propensity score matching [22]. This pattern highlights an important distinction between recurrence incidence, time-to-recurrence, and RFS. A reduction in early recurrence events may not immediately translate into improved RFS or OS, particularly in small cohorts with limited follow-up.

The largest available multicenter evidence comes from Eden et al., whose HOPE-REAL post hoc matched cohort study reported low recurrence rates in HOPE-treated HCC recipients and favorable long-term outcomes [21]. In that study, the overall recurrence rate in the HOPE-REAL HCC cohort was 6.9%, and the external matched comparison showed lower HCC recurrence in HOPE-treated recipients than in non-perfused controls. The study also identified HOPE as independently associated with improved RFS and OS. However, because the HOPE-REAL cohort included multiple European centers and may overlap with earlier single-center European studies, we treated it cautiously in quantitative synthesis. This approach avoids double-counting participants but also illustrates a central challenge in this emerging field: the strongest and most contemporary evidence may partly incorporate data from prior institutional experiences.

The biological rationale linking MP to HCC recurrence is plausible. IRI can alter both the hepatic microenvironment and systemic immune landscape, potentially facilitating tumor cell engraftment, micrometastatic outgrowth, and recurrence [5]. Proposed mechanisms include mitochondrial dysfunction, oxidative stress, release of damage-associated molecular patterns, cytokine production, adhesion molecule expression, inflammasome activation, Kupffer cell activation, neutrophil recruitment, endothelial dysfunction, and pro-angiogenic signaling [4,15,29]. MP, particularly HOPE, may reduce these processes by restoring mitochondrial function under controlled oxygenated conditions, limiting reperfusion-associated oxidative stress, and attenuating inflammatory signaling before implantation [14]. However, these mechanistic observations should be regarded as hypothesis-generating and do not demonstrate that MP causally reduces HCC recurrence in clinical practice.

Accordingly, the current clinical evidence does not support MP as an established oncologic intervention. The pooled clinical analyses in the present review did not demonstrate a statistically significant oncologic benefit. All included studies were observational, and therefore the observed associations are subject to residual confounding and cannot establish causality. MP should therefore be regarded as a graft-preservation strategy with a potential oncologic association rather than an established recurrence-modifying intervention. Donor type appears particularly important. The most favorable associations have generally been reported in settings involving DCD, extended-criteria, or otherwise higher-risk grafts, where the magnitude of IRI may be greater and therefore more modifiable [17,30]. By contrast, in lower-risk DBD settings or cohorts with already low recurrence rates, the incremental oncologic benefit of MP may be more difficult to detect. Tumor biology is also central. Microvascular invasion, tumor grade, tumor burden, AFP level, and response to locoregional therapy remain dominant determinants of recurrence, refs. [31,32,33] and any protective effect of MP is likely to act as a modifier rather than an overriding determinant of outcome.

Several sources of heterogeneity should be emphasized. First, the included studies evaluated different perfusion strategies, including HOPE, D-HOPE, and broadly coded MP in registry data. Second, comparators differed across studies, ranging from SCS to non-perfused DBD or DCD controls and external matched cohorts. Third, follow-up duration was not uniform, and early adoption of MP often resulted in shorter follow-up for perfused recipients. Fourth, recurrence ascertainment and surveillance protocols varied across centers and registries. Fifth, the degree of adjustment for tumor biology differed substantially. Some studies incorporated propensity score matching or weighting, whereas others relied on multivariable regression or comparative cohort analyses. Finally, overlap among European HOPE cohorts is possible, particularly with the HOPE-REAL dataset, requiring cautious outcome-specific synthesis.

The findings of this review extend those of a previous systematic review that evaluated heterogeneous interventions designed to reduce allograft IRI in patients undergoing LT for HCC. That review included only four retrospective studies and did not perform quantitative synthesis because of clinical and methodological heterogeneity [23]. By focusing specifically on MP and incorporating more recent clinical evidence, the present review provides a more focused quantitative assessment incorporating recently published clinical evidence. However, the limitations of the evidence base remain substantial. All included studies were observational, and no randomized trial has yet directly established whether MP reduces HCC recurrence after LT.

This review has several limitations. First, the number of included studies was small, and the primary meta-analyses were based on only a subset of studies with non-overlapping data and extractable effect estimates. Second, all included studies were non-randomized, and residual confounding by indication, donor selection, center practice, and era effects could not be excluded. Third, RFS and recurrence were not reported uniformly, and definitions of early recurrence varied across studies. Although a 2-year threshold was used as an operational framework when appropriate, heterogeneous recurrence definitions and follow-up durations may have introduced misclassification and limited direct cross-study comparison. Fourth, some registry-based data lacked detailed information on perfusion modality, tumor biology, recurrence surveillance, and perioperative management. Potentially relevant center- and transplant-level factors, including center volume, donor source, use of DCD grafts and normothermic regional perfusion, transplant selection criteria, pretransplant HCC treatment, waiting time, postoperative immunosuppression, and graft-related complications, were inconsistently reported and therefore could not be formally evaluated as sources of between-study heterogeneity. Fifth, potential cohort overlap among European HOPE studies required exclusion of some data from primary pooling, which reduced statistical power. Finally, because this review was restricted to English-language full-text articles, language bias cannot be excluded.

Future studies should aim to determine whether MP has a true causal effect on HCC recurrence or whether the observed associations reflect selection, center expertise, or improved perioperative care. Prospective validation is warranted, and the ongoing HOPE4Cancer randomized trial is expected to provide more definitive evidence [34]. In particular, adequately powered randomized controlled trials with RFS as a prespecified endpoint are needed. Such trials should be carefully designed to account for established determinants of post-transplant recurrence, including donor type, tumor burden, AFP level, Milan or expanded criteria status, response to locoregional therapy, and immunosuppression strategy. Standardized recurrence surveillance is also essential. In addition, translational endpoints should be incorporated, including perfusate biomarkers, mitochondrial injury markers, inflammatory cytokines, circulating tumor cells, circulating tumor DNA, and immune-cell profiling. These biomarkers may help identify which patients and grafts are most likely to benefit from MP as a transplant oncology strategy.

In conclusion, current clinical evidence suggests a potential favorable association between MP and oncologic outcomes after LT for HCC. However, the primary pooled estimates were not statistically significant, and the limited number of observational studies, substantial heterogeneity, and potential cohort overlap preclude definitive conclusions. Further prospective studies are needed to determine whether MP provides an oncologic benefit beyond its established role in graft preservation.

## Figures and Tables

**Figure 1:**
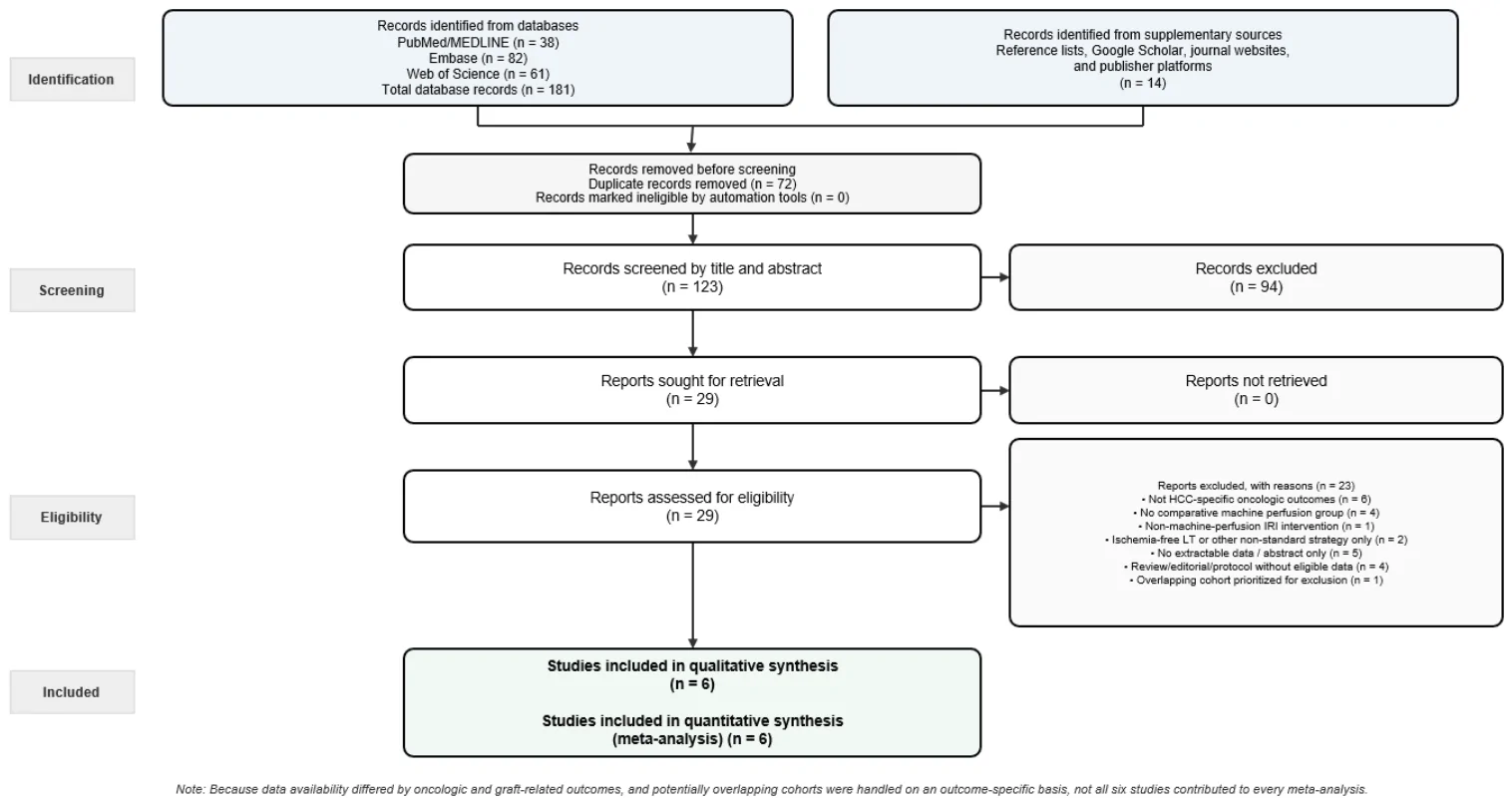
PRISMA 2020 flow diagram of study selection.

**Figure 2:**
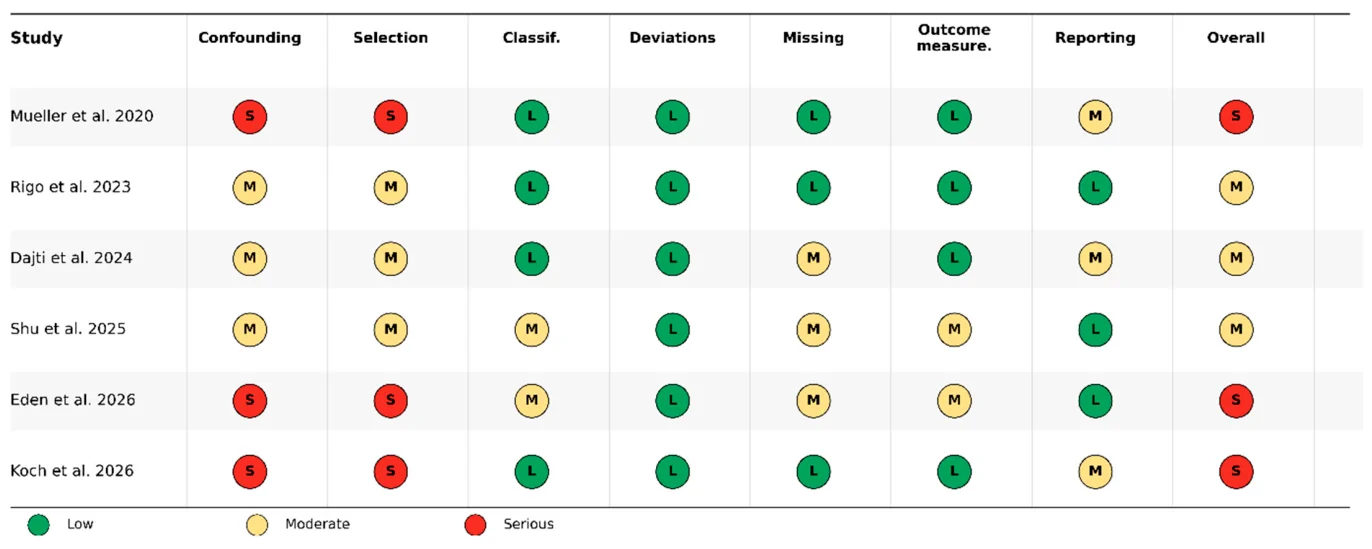
ROBINS-I risk-of-bias assessment. Studies shown: Mueller et al. [17], Rigo et al. [18], Dajti et al. [19], Shu et al. [20], Eden et al. [21], and Koch et al. [22].

**Figure 3:**
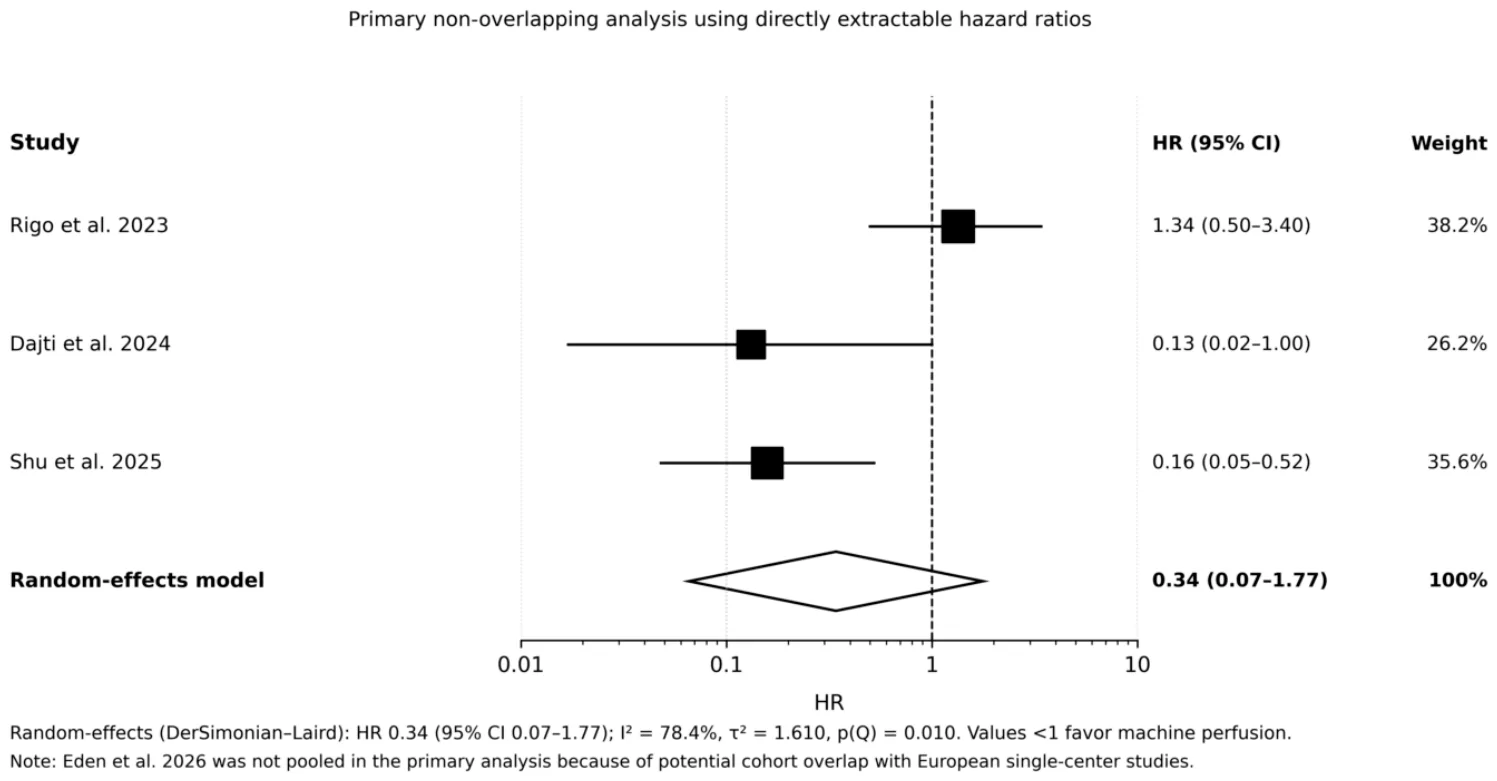
Forest plot of recurrence-free survival associated with machine perfusion after liver transplantation for hepatocellular carcinoma. Studies shown: Rigo et al. [18], Dajti et al. [19], Shu et al. [20], and Eden et al. [21].

**Figure 4:**
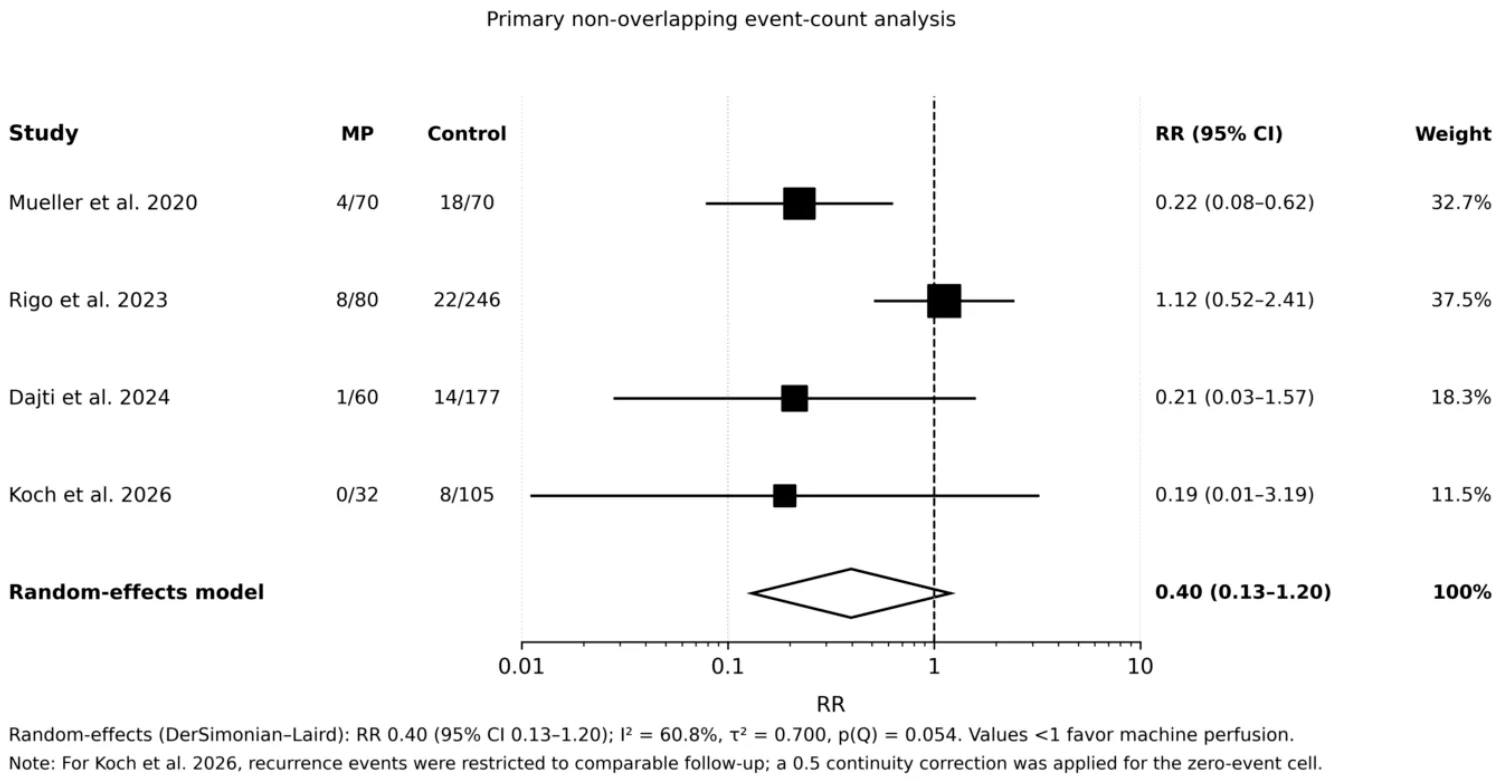
Forest plot of hepatocellular carcinoma recurrence associated with machine perfusion after liver transplantation. Studies shown: Mueller et al. [17], Rigo et al. [18], Dajti et al. [19], and Koch et al. [22].

**Table 1:** Characteristics of included studies.

Study	Country/ Data Source	Study Design and Period	Population and Comparison	Donor Type	Machine Perfusion Modality	Main Oncologic Outcomes	Adjustment/ Analytic Approach	Key Notes
Mueller et al., 2020 [17]	Two-center cohort	Retrospective comparative cohort; January 2012–September 2019	HCC recipients transplanted with HOPE-treated DCD grafts compared with non-perfused control grafts	Primarily DCD in the HOPE group; non-perfused DCD and DBD controls	End-ischemic HOPE	HCC recurrence; tumor-free survival/recurrence-free survival	Comparative cohort analysis with assessment of tumor parameters and key confounders	Early signal study suggesting lower HCC recurrence after HOPE-treated DCD LT.
Rigo et al., 2023 [18]	Italy; single center	Retrospective cohort; January 2016–December 2020	326 HCC recipients: SCS n = 246 vs. D-HOPE n = 80	D-HOPE group: DBD n = 66 and DCD n = 14; all DCD grafts treated with NRP and D-HOPE	End-ischemic D-HOPE	Recurrence-free survival; HCC recurrence	Cox regression, Bayesian model averaging, and IPTW-adjusted RFS analysis	D-HOPE reduced postoperative AST/ALT peaks but did not reduce HCC recurrence or improve adjusted RFS.
Dajti et al., 2024 [19]	Italy; single center	Retrospective cohort; 2016–2021	237 consecutive HCC recipients; HOPE n = 60 vs. SCS n = 177	DBD and DCD; HOPE: DBD n = 44, DCD n = 16; SCS: DBD n = 177	HOPE/hypothermic oxygenated perfusion	Early HCC recurrence; recurrence-free survival	Multivariable logistic regression and propensity score weighting	HOPE was associated with lower early recurrence and higher RFS after weighting.
Shu et al., 2025 [20]	United States; UNOS STAR database	Registry-based retrospective cohort; April 2015–January 2024	Adult HCC recipients in UNOS; after 1:1 PSM, MP n = 411 vs. SCS n = 411	Mixed DBD and DCD; MP group included a higher DCD proportion	Machine perfusion recorded in UNOS; modality not fully specified	Recurrence-free survival; early tumor recurrence; subgroup-defined benefit group	1:1 propensity score matching and Cox regression	Largest included study; MP was independently associated with improved RFS. Follow-up was shorter in the MP group.
Eden et al., 2026 [21]	International HOPE-REAL cohort; European multicenter data with external control cohort	Observational post hoc matched cohort; 2012–2022	599 HCC recipients receiving HOPE-treated grafts; matched comparison with non-HCC recipients and external non-perfused HCC controls	DBD n = 350; DCD n = 249	HOPE, D-HOPE, or NRP-HOPE	HCC recurrence; recurrence-free survival; overall survival	Propensity score matching	Large international cohort; potential overlap with earlier European HOPE cohorts should be assessed outcome-specifically.
Koch et al., 2026 [22]	Germany; single center	Retrospective cohort with propensity score matching	137 HCC recipients transplanted with DBD grafts: HOPE n = 32 vs. control n = 105	DBD only	HOPE	HCC recurrence; time to recurrence; recurrence-free survival; overall survival	Cox regression and propensity score matching	No recurrence was observed in the HOPE group during median 2.11-year follow-up; RFS and OS did not differ significantly.

**Abbreviations:** DBD, donation after brain death; DCD, donation after circulatory death; D-HOPE, dual hypothermic oxygenated perfusion; HCC, hepatocellular carcinoma; HOPE, hypothermic oxygenated perfusion; IPTW, inverse probability of treatment weighting; LT, liver transplantation; MP, machine perfusion; NRP, normothermic regional perfusion; NRP-HOPE, normothermic regional perfusion followed by hypothermic oxygenated perfusion; PSM, propensity score matching; RFS, recurrence-free survival; SCS, static cold storage; UNOS, United Network for Organ Sharing.

**Table 2:** Summary of oncologic outcomes in the included studies.

Study	Comparison	Follow-Up	HCC Recurrence	Recurrence-Free Survival/ Tumor-Free Survival	Overall Survival	Effect Estimate for Meta-Analysis
Mueller et al., 2020 [17]	HOPE-treated DCD grafts, n = 70, vs. non-perfused DBD controls, n = 70, at Center A; additional external non-perfused DCD and DBD cohorts, n = 70 each	Median follow-up: 2.6 years in HOPE-treated DCD group and 4.4 years in same-center DBD control group	HOPE-treated DCD: 4/70 (5.7%) vs. same-center non-perfused DBD: 18/70 (25.7%), *p* = 0.002. External controls: non-perfused DCD 10/70 (14.3%), non-perfused DBD 12/70 (17.1%)	5-year RFS: 92% vs. 73%, *p* = 0.027, favoring HOPE-treated DCD grafts	Not clearly reported as a direct comparative OS estimate for HCC groups	Binary recurrence extractable: 4/70 vs. 18/70. RFS HR not directly reported; KM reconstruction would be required if included in HR pooling.
Rigo et al., 2023 [18]	D-HOPE, n = 80, vs. SCS, n = 246	Median follow-up: 40 months in D-HOPE vs. 59 months in SCS	D-HOPE: 8/80 (10.0%) vs. SCS: 22/246 (8.9%), *p* = 0.951	1-year RFS: 95% in D-HOPE vs. 96% in SCS. Raw RFS log-rank *p* = 0.71; IPTW-adjusted RFS *p* = 0.89. Multivariable HR for D-HOPE: 1.34 (95% CI 0.50–3.40)	No direct OS benefit reported	Binary recurrence extractable: 8/80 vs. 22/246. RFS HR extractable: 1.34 (0.50–3.40).
Dajti et al., 2024 [19]	HOPE, n = 60, vs. SCS, n = 177	Median follow-up: 32 months in HOPE vs. 45 months in SCS	2-year recurrence: HOPE 1/60 (2%) vs. SCS 14/177 (8%), *p* = 0.1. After weighting, HOPE was associated with lower early recurrence: weighted OR 0.126 (95% CI 0.016–0.989), *p* = 0.049	Weighted HR for RFS: 0.132 (95% CI 0.017–0.999), *p* = 0.050	Not reported	Binary recurrence extractable: 1/60 vs. 14/177. Weighted RFS HR extractable: 0.132 (0.017–0.999).
Shu et al., 2025 [20]	UNOS cohort; after PSM, MP, n = 411, vs. SCS, n = 411	After PSM, follow-up approximately balanced: MP 12.2 months vs. SCS 12.9 months	Exact raw recurrence events not reported in the main text. Cumulative recurrence analysis showed lower recurrence in MP group; 2-year recurrence was reported as 1.7% vs. 9.3% in MP vs. SCS in the Fine–Gray analysis	2-year RFS: 96.5% in MP vs. 89.7% in SCS, *p* = 0.008. Adjusted HR for RFS: 0.158 (95% CI 0.048–0.523), *p* = 0.003	2-year OS was higher in the MP group; Cox regression showed MP as protective for OS, adjusted HR 0.316 (95% CI 0.160–0.627), *p* < 0.001	RFS HR extractable: 0.158 (0.048–0.523). Binary recurrence event-count meta-analysis not recommended unless raw events are available.
Eden et al., 2026 [21]	HOPE-REAL HCC cohort, n = 599; external matched comparison of HOPE-treated DBD HCC, n = 312, vs. non-perfused DBD HCC, n = 312	Median follow-up in HOPE-REAL cohort: 2.4 years	Overall HOPE-REAL recurrence: 41/599 (6.9%). External matched comparison: HOPE 23/312 (7.4%) vs. non-perfused 59/312 (18.9%), *p* < 0.0001	HOPE-REAL 1-, 3-, 5-year RFS: 90%, 83%, 78%. External matched comparison 5-year RFS: 77% vs. 67%, *p* = 0.019. Multivariable Cox HR for HOPE and RFS: 0.48 (95% CI 0.28–0.83), *p* = 0.008	HOPE-REAL 1-, 3-, 5-year OS: 92%, 86%, 81%. External matched comparison 5-year OS: 84% vs. 74%, *p* = 0.034. Cox HR for OS: 0.64 (95% CI 0.43–0.95), *p* = 0.030	Binary recurrence extractable: 23/312 vs. 59/312. RFS HR extractable: 0.48 (0.28–0.83). However, use as sensitivity/replacement because of likely overlap with European single-center studies.
Koch et al., 2026 [22]	HOPE, n = 32, vs. no-HOPE control, n = 105; DBD grafts only	Median follow-up: 2.11 years in HOPE vs. 7.21 years in no-HOPE	HOPE: 0/32 recurrence. No-HOPE: 15/105 (14.3%) overall; when restricted to comparable follow-up of 771 days, 8/105 (7.6%) recurred	Unmatched RFS did not differ significantly, *p* = 0.540. After PSM, RFS remained non-significant, *p* = 0.153	Unmatched OS *p* = 0.820; after PSM, OS *p* = 0.246	Binary early recurrence extractable: 0/32 vs. 8/105 using comparable follow-up. RFS HR not reported.

**Abbreviations:** DBD, donation after brain death; DCD, donation after circulatory death; D-HOPE, dual hypothermic oxygenated perfusion; HCC, hepatocellular carcinoma; HOPE, hypothermic oxygenated perfusion; HR, hazard ratio; IPTW, inverse probability of treatment weighting; KM, Kaplan–Meier; MP, machine perfusion; OS, overall survival; PSM, propensity score matching; RFS, recurrence-free survival; SCS, static cold storage; UNOS, United Network for Organ Sharing.

**Table 3:** Risk of bias assessment using the ROBINS-I tool.

Study	Confounding	Selection of Participants	Classification of Interventions	Deviations from Intended Interventions	Missing Data	Outcome Measurement	Selection of Reported Result	Overall Risk of Bias	Main Concerns
Mueller et al., 2020 [17]	Serious	Serious	Low	Low	Low	Low	Moderate	Serious	Non-randomized comparison of HOPE-treated DCD grafts with non-perfused DBD/DCD controls; center effects, donor-type differences, follow-up differences, and potential selection bias.
Rigo et al., 2023 [18]	Moderate	Moderate	Low	Low	Low	Low	Low	Moderate	D-HOPE was preferentially used for extended-criteria grafts; however, tumor characteristics and post-LT surveillance were comparable, and IPTW/BMA analyses were performed.
Dajti et al., 2024 [19]	Moderate	Moderate	Low	Low	Moderate	Low	Moderate	Moderate	Retrospective single-center design; HOPE used mainly for DCD/ECD grafts; shorter follow-up in the HOPE group; propensity score weighting performed.
Shu et al., 2025 [20]	Moderate	Moderate	Moderate	Low	Moderate	Moderate	Low	Moderate	Registry-based analysis; MP modality not fully specified; recurrence ascertainment and tumor biology variables may be limited; PSM and multivariable adjustment performed.
Eden et al., 2026 [21]	Serious	Serious	Moderate	Low	Moderate	Moderate	Low	Serious	Large post hoc multicenter cohort with external control; potential center effects, non-standardized surveillance, missing bridging/waitlist data, and possible overlap with prior European HOPE cohorts.
Koch et al., 2026 [22]	Serious	Serious	Low	Low	Low	Low	Moderate	Serious	Small single-center cohort; HOPE introduced later in the study period; marked follow-up imbalance; baseline differences in labMELD, ASA score, and bridging/downstaging therapy; PSM limited to Milan criteria status.

**Abbreviations:** ASA, American Society of Anesthesiologists; BMA, Bayesian model averaging; DBD, donation after brain death; DCD, donation after circulatory death; D-HOPE, dual hypothermic oxygenated perfusion; ECD, extended-criteria donor; HOPE, hypothermic oxygenated perfusion; IPTW, inverse probability of treatment weighting; MP, machine perfusion; PSM, propensity score matching; ROBINS-I, Risk Of Bias In Non-randomized Studies of Interventions.

## Data Availability

The data analyzed in this study were derived from published articles. The study-level data supporting the meta-analyses and sensitivity analyses are included in the Supplementary Materials. Further inquiries can be directed to the corresponding author upon reasonable request.

## Supplementary Materials

The following supporting information can be downloaded at: https://www.mdpi.com/article/10.3390/curroncol33100578/s1, Figure S1: Sensitivity analysis of recurrence-free survival including Eden et al.; Figure S2: Sensitivity analysis of HCC recurrence including Eden et al.; Table S1: Search strategies used for the systematic literature search; Table S2: Additional clinical, transplant, and perioperative characteristics of the included studies; PRISMA 2020 Checklist; Supplementary Dataset S1: Study-level data supporting the meta-analyses and sensitivity analyses.

## Abbreviations

AFP, alpha-fetoprotein; ALT, alanine aminotransferase; AST, aspartate aminotransferase; CI, confidence interval; DBD, donation after brain death; DCD, donation after circulatory death; D-HOPE, dual hypothermic oxygenated perfusion; HCC, hepatocellular carcinoma; HMP, hypothermic machine perfusion; HOPE, hypothermic oxygenated perfusion; HR, hazard ratio; IRI, ischemia–reperfusion injury; LT, liver transplantation; MP, machine perfusion; NMP, normothermic machine perfusion; OR, odds ratio; OS, overall survival; PRISMA, Preferred Reporting Items for Systematic Reviews and Meta-Analyses; PROSPERO, International Prospective Register of Systematic Reviews; RFS, recurrence-free survival; ROBINS-I, Risk Of Bias In Non-randomized Studies of Interventions; RR, risk ratio; SCS, static cold storage; UNOS, United Network for Organ Sharing.
