# Supplementary material for: Machine Perfusion and Oncologic Outcomes After Liver Transplantation for Hepatocellular Carcinoma: A Systematic Review and Meta-Analysis
Source: Curr Oncol. 2026 Sep 25;33(10):578. doi: 10.3390/curroncol33100578 (PMC13702942; doi:10.3390/curroncol33100578)
Supplement: Supplementary file 1 [file curroncol-33-00578-s001.zip › mpHCCSRMAcurrentoncosupplementarymaterials/PRISMA2020ChecklistmpHCCSRMA.pdf]

PRISMA 2020 Checklist

Machine Perfusion and Oncologic Outcomes After Liver Transplantation for Hepatocellular Carcinoma: A Systematic Review and Meta-Analysis

Page numbers refer to the submitted manuscript.

Section	Item	PRISMA 2020 checklist item	Location
TITLE	1	Identify the report as a systematic review.	1
ABSTRACT	2	See the PRISMA 2020 for Abstracts checklist.	3
INTRODUCTION	3	Describe the rationale for the review in the context of existing knowledge.	5–6
	4	Provide an explicit statement of the objective(s) or question(s) the review addresses.	6
METHODS	5	Specify the inclusion and exclusion criteria for the review and how studies were grouped for the syntheses.	7
	6	Specify all databases, registers, websites, organisations, reference lists and other sources searched or consulted to identify studies. Specify the date when each source was last searched or consulted.	7–8
	7	Present the full search strategies for all databases, registers and websites, including any filters and limits used.	8; Supplementary Table S1
	8	Specify the methods used to decide whether a study met the inclusion criteria, including how many reviewers screened each record/report, whether they worked independently, and how disagreements were resolved.	8
	9	Specify the methods used to collect data from reports, including how many reviewers collected data from each report, whether they worked independently, any processes for obtaining or confirming data from study investigators, and, if applicable, details of automation tools used.	8
	10a	List and define all outcomes for which data were sought. Specify whether all results compatible with each outcome domain in each study were sought and, if not, the methods used to decide which results to collect.	7, 8–9
	10b	List and define all other variables for which data were sought. Describe any assumptions made about any missing or unclear information.	8
	11	Specify the methods used to assess risk of bias in the included studies, including details of the tool(s) used, how many reviewers assessed each study and whether they worked independently.	9
	12	Specify for each outcome the effect measure(s) used in the synthesis or presentation of results.	8–9
	13a	Describe the processes used to decide which studies were eligible for each synthesis.	8–10
	13b	Describe any methods required to prepare the data for presentation or synthesis, such as handling of missing summary statistics or data conversions.	8–9
	13c	Describe any methods used to tabulate or visually display results of individual studies and syntheses.	9; Figures 3–4; Supplementary Figures S1–S2
	13d	Describe any methods used to synthesize results and provide a rationale for the choice(s). If meta-analysis was performed, describe the model(s), method(s) to identify statistical heterogeneity, and software package(s) used.	9
	13e	Describe any methods used to explore possible causes of heterogeneity among study results.	10
	13f	Describe any sensitivity analyses conducted to assess robustness of the synthesized results.	10
	14	Describe any methods used to assess risk of bias due to missing results in a synthesis (arising from reporting biases).	9
	15	Describe any methods used to assess certainty (or confidence) in the body of evidence for an outcome.	Not formally assessed
RESULTS	16a	Describe the results of the search and selection process, from the number of records identified in the search to the number of studies included in the review, ideally using a flow diagram.	11; Figure 1
	16b	Cite studies that might appear to meet the inclusion criteria, but which were excluded, and explain why they were excluded.	11 (categories and reasons for full-text exclusions)
	17	Cite each included study and present its characteristics.	11–12; Table 1
	18	Present assessments of risk of bias for each included study.	12; Figure 2; Table 3
	19	For all outcomes, present, for each study: summary statistics for each group (where appropriate) and an effect estimate and its precision, preferably using structured tables or plots.	12–16; Table 2; Figures 3–4
	20a	For each synthesis, briefly summarise the characteristics and risk of bias among contributing studies.	12–16; Tables 1–3
	20b	Present results of all statistical syntheses conducted. If meta-analysis was done, present for each the summary estimate and its precision and measures of statistical heterogeneity. If comparing groups, describe the direction of the effect.	12–14; Figures 3–4
	20c	Present results of all investigations of possible causes of heterogeneity among study results.	13–14; 17–18
	20d	Present results of all sensitivity analyses conducted to assess robustness of the synthesized results.	13–14; Supplementary Figures S1–S2
	21	Present assessments of risk of bias due to missing results (arising from reporting biases) for each synthesis assessed.	14 (not formally assessed because <10 studies per pooled analysis)
	22	Present assessments of certainty (or confidence) in the body of evidence for each outcome assessed.	Not formally assessed
DISCUSSION	23a	Provide a general interpretation of the results in the context of other evidence.	17–19
	23b	Discuss any limitations of the evidence included in the review.	18
	23c	Discuss any limitations of the review processes used.	18
	23d	Discuss implications of the results for practice, policy, and future research.	18–19
OTHER INFORMATION	24a	Provide registration information for the review, including register name and registration number, or state that the review was not registered.	1, 7 (PROSPERO CRD420261406720)
	24b	Indicate where the review protocol can be accessed, or state that a protocol was not prepared.	7 (PROSPERO registration; protocol availability not otherwise stated in manuscript)
	24c	Describe and explain any amendments to information provided at registration or in the protocol.	Not reported / no amendments stated
	25	Describe sources of financial or non-financial support for the review, and the role of the funders or sponsors in the review.	2 (no external funding)

Section	Item	PRISMA 2020 checklist item	Location
	26	Declare any competing interests of review authors.	2 (no conflict of interest)
	27	Report which of the following are publicly available and where they can be found: template data collection forms; data extracted from included studies; data used for all analyses; analytic code; any other materials used in the review.	2; Supplementary Materials (study-level data supporting meta-analyses and sensitivity analyses)

Note: Items 15 and 22 were not formally assessed because the manuscript does not use a formal certainty-of-evidence framework (e.g., GRADE). Items 24b–24c are reported conservatively based on the manuscript text.
