# Supplementary material for: Machine Perfusion and Oncologic Outcomes After Liver Transplantation for Hepatocellular Carcinoma: A Systematic Review and Meta-Analysis
Source: Curr Oncol. 2026 Sep 25;33(10):578. doi: 10.3390/curroncol33100578 (PMC13702942; doi:10.3390/curroncol33100578)
Supplement: Supplementary file 1 [file curroncol-33-00578-s001.zip › mpHCCSRMAcurrentoncosupplementarymaterials/tableS1searchstrategymphcc.pdf]

Supplementary Table S1. Search strategies used for the systematic literature search

| Information source | Search strategy |
|---|---|
| **PubMed/MEDLINE** | ("Liver Transplantation"[Mesh] OR "liver transplantation"[Title/Abstract] OR "liver transplant"[Title/Abstract] OR "liver transplants"[Title/Abstract] OR "liver transplanted"[Title/Abstract] OR "hepatic transplantation"[Title/Abstract] OR "hepatic transplant"[Title/Abstract] OR "hepatic transplants"[Title/Abstract]) AND ("Carcinoma, Hepatocellular"[Mesh] OR "Liver Neoplasms"[Mesh] OR "hepatocellular carcinoma"[Title/Abstract] OR HCC[Title/Abstract] OR "liver cancer"[Title/Abstract] OR "hepatic cancer"[Title/Abstract] OR "primary liver cancer"[Title/Abstract]) AND ("machine perfusion"[Title/Abstract] OR "ex vivo perfusion"[Title/Abstract] OR "ex situ perfusion"[Title/Abstract] OR "normothermic machine perfusion"[Title/Abstract] OR NMP[Title/Abstract] OR "hypothermic machine perfusion"[Title/Abstract] OR HMP[Title/Abstract] OR "hypothermic oxygenated perfusion"[Title/Abstract] OR HOPE[Title/Abstract] OR "D-HOPE"[Title/Abstract] OR DHOPE[Title/Abstract] OR "dual hypothermic oxygenated perfusion"[Title/Abstract] OR "oxygenated machine perfusion"[Title/Abstract] OR "subnormothermic machine perfusion"[Title/Abstract] OR SNMP[Title/Abstract] OR "normothermic regional perfusion"[Title/Abstract] OR NRP[Title/Abstract] OR "ischemia-free liver transplantation"[Title/Abstract] OR "ischemia free liver transplantation"[Title/Abstract]) AND english[Language] |
| **Embase, Ovid** | 1. exp liver transplantation/ OR (liver transplantation OR liver transplant* OR hepatic transplantation OR hepatic transplant*).ti,ab,kw. 2. exp hepatocellular carcinoma/ OR exp liver tumor/ OR exp liver cancer/ OR (hepatocellular carcinoma OR HCC OR liver cancer OR hepatic cancer OR primary liver cancer).ti,ab,kw. 3. exp machine perfusion/ OR exp organ preservation/ OR (machine perfusion OR ex vivo perfusion OR ex situ perfusion OR normothermic machine perfusion OR NMP OR hypothermic machine perfusion OR HMP OR hypothermic oxygenated perfusion OR HOPE OR D-HOPE OR DHOPE OR dual hypothermic oxygenated perfusion OR oxygenated machine perfusion OR subnormothermic machine perfusion OR SNMP OR normothermic regional perfusion OR NRP OR ischemia-free liver transplantation OR ischemia free liver transplantation).ti,ab,kw. 4. 1 AND 2 AND 3 5. limit 4 to English language 6. 5 NOT (exp animal/ NOT exp human/) |

| Information source | Search strategy |
| --- | --- |
| **Web of Science Core Collection** | TS=(("liver transplantation" OR "liver transplant*" OR "hepatic transplantation" OR "hepatic transplant*") AND ("hepatocellular carcinoma" OR HCC OR "liver cancer" OR "hepatic cancer" OR "primary liver cancer") AND ("machine perfusion" OR "ex vivo perfusion" OR "ex situ perfusion" OR "normothermic machine perfusion" OR NMP OR "hypothermic machine perfusion" OR HMP OR "hypothermic oxygenated perfusion" OR HOPE OR "D-HOPE" OR DHOPE OR "dual hypothermic oxygenated perfusion" OR "oxygenated machine perfusion" OR "subnormothermic machine perfusion" OR SNMP OR "normothermic regional perfusion" OR NRP OR "ischemia-free liver transplantation" OR "ischemia free liver transplantation")) Refined by: Languages = English |
| **Supplementary manual search** | Manual searches were performed in Google Scholar, relevant journal websites, publisher platforms, and the reference lists of included studies and relevant reviews. The following keyword combinations were used: "hepatocellular carcinoma" "liver transplantation" "machine perfusion" recurrence "HCC" "liver transplantation" "machine perfusion" "recurrence-free survival" "hypothermic oxygenated perfusion" "hepatocellular carcinoma recurrence" "liver transplantation" "HOPE" "HCC recurrence" "liver transplantation" "dual hypothermic oxygenated perfusion" "HCC" "liver transplantation" "normothermic machine perfusion" "hepatocellular carcinoma" "liver transplantation" "machine perfusion" "oncologic outcomes" "liver transplantation" "hepatocellular carcinoma" Recently published full-text articles identified through publisher platforms or journal websites were considered eligible if they met the predefined inclusion criteria and had sufficient extractable data, even if they were not yet indexed in PubMed/MEDLINE at the time of the final search. |

**Abbreviations:** D-HOPE, dual hypothermic oxygenated perfusion; HCC, hepatocellular carcinoma; HMP, hypothermic machine perfusion; HOPE, hypothermic oxygenated perfusion; NMP, normothermic machine perfusion; NRP, normothermic regional perfusion; SNMP, subnormothermic machine perfusion.

**Supplementary Table S1 note:** Terms related to normothermic regional perfusion and ischemia-free liver transplantation were included to maximize retrieval of mechanistically related studies. However, these studies were assessed separately according to the predefined eligibility criteria. Strict

ex situ machine perfusion studies were included in the primary analysis, whereas ischemia-free liver transplantation and other non-standard ischemia-reduction strategies were considered for narrative or sensitivity analyses only.
