# Supplementary material for: Machine Perfusion and Oncologic Outcomes After Liver Transplantation for Hepatocellular Carcinoma: A Systematic Review and Meta-Analysis
Source: Curr Oncol. 2026 Sep 25;33(10):578. doi: 10.3390/curroncol33100578 (PMC13702942; doi:10.3390/curroncol33100578)
Supplement: Supplementary file 1 [file curroncol-33-00578-s001.zip › mpHCCSRMAcurrentoncosupplementarymaterials/tableS2additionalclinicalcharacteristics.pdf]

Supplementary Table S2. Additional clinical, transplant, and perioperative characteristics of the included studies.

Study	Center setting / volume	Donor source and NRP	HCC selection / listing criteria	Pre-LT HCC treatment and waiting time	HAT / PNF / biliary complications	ICU / hospital stay	Post-LT immunosuppression
**Mueller et al. (2020) [17]**	Two centers: University Hospital Zurich (Center A) and Queen Elizabeth Hospital Birmingham (Center B). Annual LT center volume NR.	Deceased-donor grafts: DCD-HOPE at Center A; unperfused DCD and DBD control cohorts at Centers A/B. NRP not reported.	Milan, UCSF, and Metroticket 2.0 status were documented. Center B followed Milan criteria strictly during the study period.	Bridging treatment: 65/70 (92.9%) DCD-HOPE A; 35/70 (50.0%) DCD B; 64/70 (91.4%) DBD A; 41/70 (58.6%) DBD B. Median waiting time (days), same order: 155.5; 63; 263; 70.	HAT: NR. PNF: NR. Biliary complications: NR.	Median ICU stay (days): 3 [2–4.25]; 3 [2–6]; 2 [1–4]; 2 [2–4.25]. Median hospital stay (days): 17 [13–22]; 9 [7–15]; 15 [12–24]; 8 [7–14] (same cohort order as preceding column).	Center A: tapered steroids + tacrolimus; basiliximab used with delayed tacrolimus in impaired renal function. Center B: steroids + tacrolimus + azathioprine or mycophenolate mofetil.
**Rigo et al. (2023) [18]**	Single center (Turin, Italy). Annual LT center volume NR.	D-HOPE: 66 DBD + 14 DCD (1 Maastricht II, 13 Maastricht III); SCS: 246 DBD. All DCD grafts underwent sequential NRP followed by D-HOPE.	Exact listing policy NR. Tumor burden/AFP were recorded and Metroticket 2.0 was used to estimate 5-year survival probability.	D-HOPE vs SCS: downstaging 79% vs 82%; locoregional therapy 70% vs 78%; SBRT 18% vs 8%; prior liver resection 4% vs 5%. Median waiting time 32 [14.5–94.5] vs 35 [17–86] days.	HAT: NR. PNF: NR. Biliary complications 18% vs 20%; ischemic cholangiopathy 0% vs 3% (D-HOPE vs SCS).	Median ICU stay 3 [2–5] vs 3 [2–4] days; hospital stay 11 [8–18] vs 10 [8–14.8] days (D-HOPE vs SCS).	Basiliximab induction, tacrolimus, steroids, and mycophenolate mofetil; steroids tapered off by 3 months; everolimus considered from 1 month. Basiliximab: 54% vs 20%; everolimus use: 71% vs 71% (D-HOPE vs SCS).
**Dajti et al. (2024) [19]**	Single center (Policlinico Sant'Orsola, Bologna, Italy). Annual LT center volume NR.	HOPE: 16/60 DCD (27%) and 44 DBD; SCS: 177 DBD. NRP not reported.	National ISO score 2.0 used for HCC list prioritization from 2019 (MELD-Na, response to previous HCC treatment, and waiting-list time); Milan status reported at listing and before LT.	Pre-LT HCC therapy in 90% overall. HOPE vs SCS: RFA 45% vs 52%; TACE 62% vs 51%; liver resection 23% vs 17%; other 13% vs 25%. Waiting time not reported numerically.	HAT: NR. PNF 1/60 (2%) vs 2/177 (1%) (HOPE vs SCS). Biliary complications: NR.	ICU stay: NR. Median hospital stay 15 [12–20] vs 14 [11–20] days (HOPE vs SCS).	NR.
**Shu et al. (2025) [20]**	UNOS/STAR national registry cohort; center-specific volume NR.	DCD status reported; after PSM, DCD donors were 199/411 (48.4%) in MP and 205/411 (49.9%) in SCS. Living vs deceased donor status and NRP use were not separately reported in the article.	Milan status reported (within Milan after PSM: 83.9% MP vs 82.7% SCS). Specific center listing policies/expanded criteria NR.	Pre-LT bridging/downstaging treatment NR. Median waiting time after PSM: 257 [188.5–454.5] vs 242 [185–351] days (MP vs SCS).	HAT: NR. PNF: NR. Biliary complications: NR.	ICU stay: NR. Hospital stay: NR.	NR.
**Eden et al. (2026) [21]**	HOPE-REAL: 22 European LT centers. Participating centers were required to have performed ≥20 HOPE procedures; annual LT center volume NR.	599 HCC recipients: 350 DBD and 249 DCD. HOPE 273/599 (45.6%), D-HOPE 235/599 (39.2%), NRP-HOPE 91/599 (15.2%; 36.5% of DCD cases).	Milan criteria and AFP model used for oncologic risk stratification. Center-specific LT listing criteria were not standardized/reported.	Waiting time and potential bridging therapies were explicitly unavailable retrospectively across all centers.	HAT: NR. PNF: NR. Biliary complications: NR.	HOPE-REAL HCC cohort overall: median ICU stay 4 [2–6] days; hospital stay 15 [11–21] days. Comparative values versus the external non-perfused HCC control were not reported.	mTOR inhibitor use 12/599 (2%); detailed post-LT immunosuppression protocol NR.
**Koch et al. (2026) [22]**	Single center (LMU University Hospital Munich, Germany). Annual LT center volume NR.	All 137 grafts were DBD. NRP not applicable to this DBD-only cohort.	LT required approval by national tumor and transplant boards. Milan status was analyzed and used for PSM but was not stated as an absolute inclusion criterion.	Bridging/downstaging therapy: 71.9% HOPE vs 90.5% no-HOPE. Waiting time NR.	HAT: NR. PNF: NR. Biliary complications: NR.	ICU stay: NR. Hospital stay: NR.	Tacrolimus + mycophenolate mofetil + corticosteroids; some patients were switched to mTOR inhibitors (e.g., everolimus).

Abbreviations: DBD, donation after brain death; DCD, donation after circulatory death; D-HOPE, dual hypothermic oxygenated perfusion; HAT, hepatic artery thrombosis; HOPE, hypothermic oxygenated perfusion; ICU, intensive care unit; LT, liver transplantation; MP, machine perfusion; N/A, not applicable; NRP, normothermic regional perfusion; NR, not reported in the source article; PNF, primary nonfunction; PSM, propensity score matching; RFA, radiofrequency ablation; SBRT, stereotactic body radiotherapy; SCS, static cold storage; TACE, transarterial chemoembolization.
